# Ethical perspectives of certified public accountants and the cannabis industry

**DOI:** 10.1186/s42238-022-00118-z

**Published:** 2022-01-26

**Authors:** G. Suzanne Owens-Ott, Johnny Snyder, Richard Ott

**Affiliations:** Colorado Mesa University, Grand Junction, CO USA

**Keywords:** CPA, Cannabis, Cannabis industry, Accounting, Ethics, Certified public accounting

## Abstract

**Background:**

Certified public accountants must follow very high standards of ethical conduct as set forth by the AICPA Code of Professional Conduct and individual state licensing requirements. A 2019 grounded theory qualitative study posed that CPAs remain largely hesitant to serve the cannabis industry primarily because they fear federal prosecution as long as cannabis remains on the DEA’s Schedule I Drug List. The purpose of this research was to determine the perceptions of CPAs regarding providing accounting services to the cannabis industry in states that have legalized cannabis usage. This study investigated whether CPAs would serve the industry, why they might decline to serve the industry, what risks they believe serving the industry posed, and whether they believe serving the cannabis industry would create a moral or ethical issue.

**Methods:**

This follow-up quantitative study investigated a small convenience sample of approximately one hundred CPAs in Colorado and Washington to learn more about their perceptions of serving the cannabis industry. Data was analyzed using chi-square and Mann-Whitney *U* tests to determine if there were any differences in perceptions between groups such as states, gender, and age categories.

**Results:**

Of the participants, 77% responded that neither they nor their firm provided services to a cannabis-related business client compared to 23% that did serve cannabis clients. More Colorado CPAs were willing to turn down CRB work than were expected and fewer Colorado CPAs would be willing to take on CRB clients than were expected. While in Washington, fewer CPAs would turn down RB clients than expected, and more are willing to accept CRB clients than were expected. The risk due to potential liability coverage issues due to serving the cannabis industry was rated the highest while the risk of losing the CPA license was rated lowest. Data indicated that there was not a statistically significant difference between Colorado and Washington participants related to whether they were morally or religiously opposed to working in the industry or if they viewed serving the industry as an ethical violation.

**Conclusion:**

CPAs remain largely unwilling to serve the cannabis industry primarily because CPAs fear federal prosecution as long as cannabis remains on the DEA’s Schedule I Drug Listing. The results of this study indicate that while most CPAs are not morally or religiously opposed to serving the industry, about half still believe doing so may constitute an ethical violation for a CPA.

## Introduction

Certified public accountants (CPAs) must follow the Code of Professional Conduct set forth by the American Institute of Certified Public Accountants (AICPA) and individual state codes of conduct. Because of this, the CPA profession is believed to require an extremely high standard of ethical conduct (AICPA 2014). A “lack of good moral character” could be interpreted as a violation of ethics and result in the loss of a CPA’s license to practice which could damage a CPA’s ability to earn a living (AICPA 2016, p. 11). As a result of these high ethical standards, many CPAs are hesitant, if not completely unwilling, to provide services to the cannabis industry even in states where cannabis has been legalized for medical or recreational use. This quantitative study investigated CPAs’ ethical perceptions of serving the cannabis industry in Colorado and Washington, the two states with the most mature cannabis laws and industries.

## Background

Currently, eleven states plus the District of Columbia and the Northern Mariana Islands have legalized medical and recreational cannabis while thirty-three states plus the District of Columbia, Puerto Rico, the U.S. Virgin Islands, and Guam have legalized medical cannabis. The number of states with legalized cannabis is projected to grow in the coming years (National Conference of State Legislatures 2020). However, cannabis remains illegal and classified as a Schedule I controlled substance at the federal level (Robinson 2017). Throughout the USA, there remains a dichotomy of viewpoints regarding cannabis. Some Americans believe cannabis is a dangerous drug, due to its psychoactive properties, that will lead to violence and crime and should remain outlawed (Cheon et al. 2018). Many Americans believe that cannabis is a gateway drug that will lead individuals to use other, harder drugs (Doherty et al. 2015), while a contingent of Americans see cannabis as having potential medical and industrial benefits that outweigh any danger to society (Pew Research Center 2015). Others view cannabis as less dangerous, or at least no more dangerous, than alcohol and believe the drug should be legalized for use by adults (Pew Research Center 2015). These differences of opinion continue to be debated in the legal system and, particularly, in the political arena (Pew Research Center 2015). This conflict between federal and state laws, as well as the dichotomy of viewpoints, creates the business problem addressed by this study.

Because of federal illegality, cannabis-related businesses (CRBs), even when operating legally under state laws, may have difficulty obtaining professional financial services (Sterna and Wolfe 2017). For example, CRBs face difficulty in obtaining banking services such as checking, credit cards, electronic transfers, and loans (Taylor et al. 2016). Many certified public accountants may be unwilling to provide their accounting and tax services to CRBs due to increased risks associated with the industry (AICPA 2016; Taylor et al. 2016).

### Purpose of the study

The purpose of this research was to determine the perceptions of CPAs regarding the provision of accounting services to the cannabis industry in states that have legalized cannabis for recreational use. The two most mature cannabis markets are in Washington and Colorado and include producers, processors, and retailers. Both states have cannabis industries consisting of many small businesses; in fact, 42% of the Colorado market comes from “corporations that would be considered a small business by the U.S. Small Business Administration (less than $8 million for specialty retail stores)” (MPG Consulting and Leeds School of Business 2019, page 34). CPAs in Colorado and Washington were surveyed to gain information regarding their willingness to serve the cannabis industry and their perceptions regarding the ethical issues of doing so. This study was needed to determine if and why CPAs are unwilling to serve the industry. The following research questions were studied:

1. Will CPAs serve the cannabis industry?
2. Why are CPAs unwilling to provide services to cannabis-related businesses?
3. What do CPAs believe is the primary risk related to serving the cannabis industry?
4. Do CPAs believe serving the cannabis industry would create an ethical issue?

Accounting professionals likely have a myriad of reasons for not servicing the cannabis industry. CPAs may be unwilling to provide the services due to conflicting federal and state regulations which would result in a violation of federal law and potential criminal charges. CPAs may also be unwilling to provide the services for fear of violating professional ethics codes and losing their professional permit to practice which could potentially reduce or eliminate the CPA’s ability to earn a living. Some CPAs or CPA firms may elect to decline the business because of an individual moral objection to participating in the cannabis industry. Lastly, some CPAs may choose to avoid the cannabis industry due to the extensive learning required to obtain the specialized knowledge required to serve the complex, cash-intensive, and high-risk industry efficiently and effectively.

### Literature review

CPAs are granted a license to practice from the state(s) in which they provide services (AICPA 2016). An ethical infraction or “lack of good moral character” may cause the CPA to lose his or her license to practice (AICPA 2016, p. 11). The National Association of State Boards of Accountancy cautions CPAs to verify with their state accountancy boards as to whether providing services to the cannabis industry would constitute “an ‘act discreditable’” (NASBA 2019, para. 2). Because cannabis business violates federal law, the provision of services by a CPA related to a cannabis business could potentially be considered a violation of good moral character or an act discreditable (NASBA 2019).

State boards of accountancy have not been entirely clear in their official guidance to CPAs on the provision of services for the cannabis industry (AICPA 2016). For example, the Washington State Board of Accountancy (BOA) states that provision of services to a cannabis-related business does not constitute a violation of the BOA’s rules (Satterlund 2018). However, the Washington BOA further recommended that CPAs consider the risks associated with serving the cannabis industry and that CPAs engage an attorney for counsel (Satterlund 2018). The 2018 statement by the Washington BOA followed the March 2018 signing of the Washington State Engrossed Substitute Senate Bill 5928 which states:

A certified public accountant or certified public accounting firm, which practices public accounting as defined in RCW 18.04.025, does not commit a crime solely for providing professional accounting services as specified in RCW 18.04.025 for a marijuana producer, marijuana processor, or marijuana retailer authorized under chapter 69.50 RCW (Satterlund 2018, para. 3).

In Colorado, the BOA issued a Position Statement that indicated the CPA’s provision of services to the cannabis industry is not “specifically prohibited by the Accountancy Act” (Colorado Board of Accountancy 2015, para. 1). The Colorado Board went on to caution CPAs that this:

should not be construed: (a) as an endorsement for certificate holders to provide professional services to the marijuana industry; (b) as a statement about the feasibility of meeting applicable professional standards in providing services to the marijuana industry; or (c) as a statement about marijuana enforcement in any other jurisdiction or by any other local, state, or federal authority. (Colorado Board of Accountancy 2015, para. 3)

The provision of services to cannabis businesses may not necessarily constitute a violation of good moral character, but other issues can arise from a CPA servicing the cannabis industry (Sterna and Wolfe 2017). For example, a CPA may be judged to have “aided and abetted” or been involved in a “conspiracy to violate” the federal Controlled Substances Act or racketeering laws (Sterna and Wolfe 2017, p. 9). Participating in “dishonest, fraudulent, or criminal acts” associated with the cannabis industry may result in the CPA being exposed to criminal prosecution that could result in fines, penalties, or other discipline (Sterna and Wolfe 2017, p. 10).

New Mexico adopted a law that licensed CRBs would need to have financial statements audited by a CPA to submit to the governing authority (Chiang et al. 2019). The New Mexico Board of Accountancy (NMBOA), however, did not issue guidance to licensed CPAs indicating approval of this service, and therefore, no CPAs would do the work (Chiang et al. 2019). CPAs are strongly encouraged by the state boards that have specifically addressed serving the cannabis industry, the AICPA, and NASBA to proceed with caution and seek legal counsel when entering the cannabis industry (Chiang et al. 2019; NASBA 2019).

The findings from a 2019 qualitative study supported the belief that many CPAs are not willing to serve the cannabis industry primarily because the drug remains federally illegal (Owens-Ott 2020). However, that study found a small number of CPAs are willing to serve the cannabis industry which means that CRBs do not have to find substitutes for professional CPA services (Owens-Ott 2020). That study further determined that to competently serve the cannabis industry, CPAs need to acquire significant industry experience and have a thorough knowledge of state and local regulations, U.S. Tax Code § 280E: “Expenditures in connection with the illegal sale of drugs,” and internal controls for a cash-intensive business (Owens-Ott 2020).

In addition, CPAs may believe there is reputational risk with current or prospective clients in associating with the cannabis industry. Reputation of a firm could potentially be seen by outsiders as an indication of the firm’s quality of services (Devers et al. 2009). CPA firms may be believed to be less than legitimate based on their association with the somewhat controversial cannabis industry (Devers et al. 2009). A core stigmatized organization is one for whom outsiders have a “perceived violation of social norms,” and these outsiders may look at the organization unfavorably (Hudson and Okhuysen 2009, p.134). Current or prospective clients may avoid engaging with a CPA who works in the cannabis industry because they worry that a negative stigma may transfer to them (Hampel and Tracey 2016). Some CPAs may strategize that they are willing to accept such core stigma as part of their business as may be the case for CPA firms who specialize in cannabis clients (Hudson and Okhuysen 2009).

Because qualitative research based on interviews provides the information “filtered through the view of interviewees,” the perspectives of the participants of the Owens-Ott (2020) study may not necessarily be reflective of all CPAs (Creswell and Creswell 2018, p. 188). In addition, many of the participants in the qualitative study were found through the researcher’s professional network thus their responses might not be as “equally articulate and perceptive” as other participants might be (Creswell and Creswell 2018, p. 188). Finally, as is the case with most qualitative research, the number of participants in the Owens-Ott (2020) study was limited due to the large amount of in-depth interview data that had to be collected. To expand knowledge in this field of study and the findings of the qualitative study, this research project was initiated. Using the findings from the Owens-Ott (2020) study, this project was designed to obtain data from a larger sample size than was possible with the qualitative study.

## Methodology

### Participants

This study surveyed CPAs in the states of Colorado and Washington, the two states with the most mature cannabis markets. A few of the participants were from other states including California or were licensed in multiple states in addition to Colorado or Washington; these responses were not included in the data analysis. Prior to contacting any potential participants, the study was approved by university’s Institutional Review Board (IRB). All participants were over 21 years of age and were required to agree to a statement of informed consent that explained the study, how they would be protected, and any risks associated with participation. Prior to opening the survey, participants had to agree to the statement of informed consent.

Potential CPA participants were identified from the listing of CPA firms from the Colorado and Washington State Boards of Accountancy websites. Using this list, CPA firms’ websites were reviewed to find individual CPA email addresses to contact to request participation in the study. The researcher emailed 885 participation requests to CPAs in Colorado and Washington. The survey request was also posted on LinkedIn and Facebook on which the author has numerous CPA contacts. A total of 101 surveys were collected, representing a 11.4% return rate of the emails that were sent out. It is unknown how many survey respondents originated from the email requests versus the social media posts.

### Instrumentation

Developing the survey to support the research questions necessitated several iterations (Miles and Huberman 1994). While writing the survey, the researchers followed the advice of Thomas (2009) by keeping the survey brief, clear, and precise and by including all needed details and avoiding “prestige bias” (Thomas 2009, p. 174). A copy of the survey can be viewed in Appendix. The survey consisted of multiple choice, rank order, rating scale, Likert Scale, and open-ended questions. Filters were used to move participants to the next, relevant question depending on their answer to previous question (Thomas 2009).

### Data collection

SurveyMonkey, a cloud-based survey tool, was used to administer the survey. Participants were provided a link to the survey in SurveyMonkey and asked to complete the survey. All 101 participants completed the survey using SurveyMonkey. To protect the participants’ privacy, no identifying information was collected in the survey.

### Data analysis

Some of the data collected are demographic and are used to segment the analysis and yield context to other data collected (Polonsky and Waller 2005). The authors compared the data collected, particularly the responses to open-ended questions, to the themes and patterns identified in the original Owens-Ott 2020 qualitative study (Thomas 2009). Themes and patterns in the original study included accountants’ unwillingness to serve the cannabis industry mostly because of fear of federal prosecution and potential loss of the CPA license. Data was analyzed to determine if there were any differences in perceptions between groups such as states, gender, and age categories. Triangulation was used to compare data collected from both qualitative and quantitative questions in this study as well as from the previous qualitative study conducted by the Owens-Ott study in order to increase the “confidence in the findings” (Bryman and Bell 2011, p. 631).

## Results

**Fig. 1:**
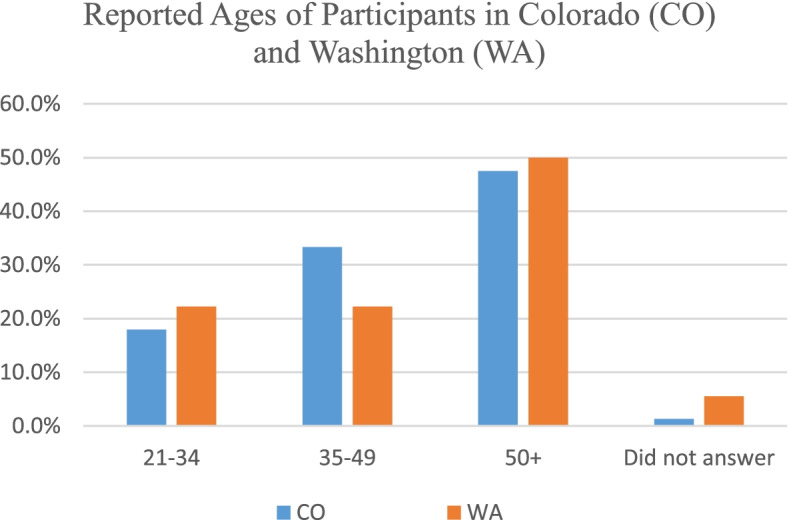
Ages of certified public accountants (CPAs) responding to survey

In total, 101 participants agreed to the Informed Consent and opened the survey. After cleansing the data for CPAs who are not licensed in Colorado or Washington, a data set with *n* = 96 CPAs was obtained. The results represent an averaging of the proportions of respondents. The number of participants that responded to the survey is relatively small considering there are nearly 39,000 active individual CPA licensees according to the two states’ Board of Accountancy websites as of November 29, 2021. This data set included 70.8% male and 28.1% female with 1% who preferred not to identify gender. Figure 1 shows the reported ages of the participants by age group.

**Fig. 2:**
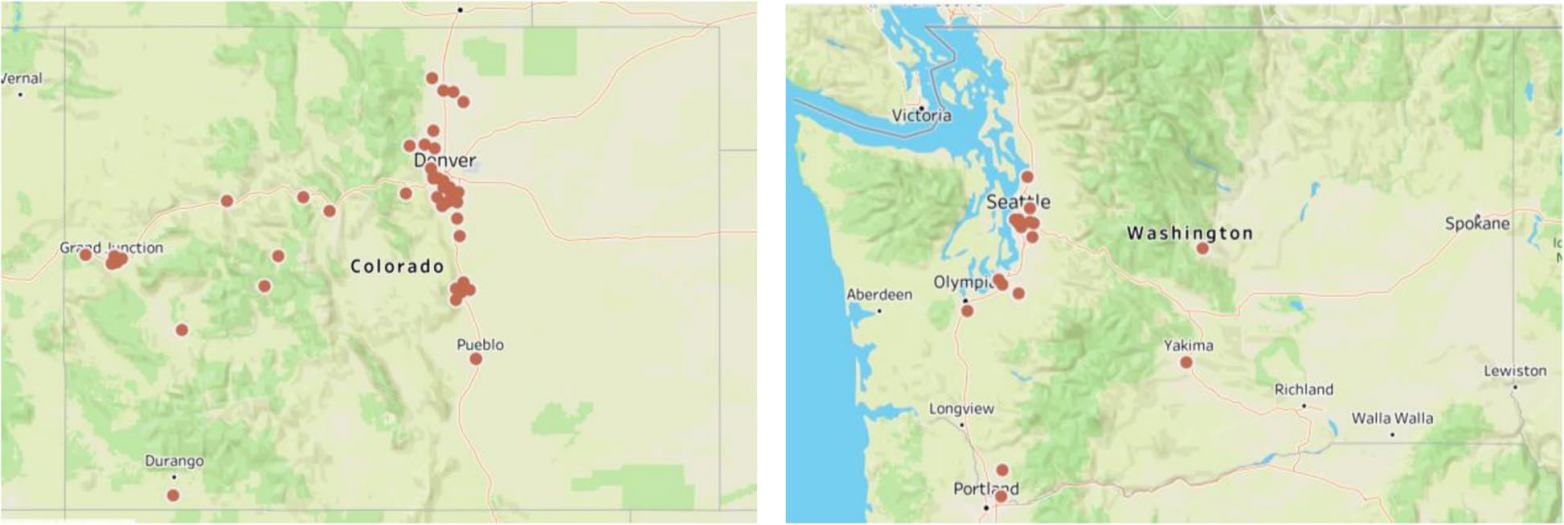
Location of participants by zip code in Colorado and Washington

A graph of their locations is given in Fig. 2, where it can be seen that most survey respondents come from urban areas along major transportation corridors.

**Fig. 3:**
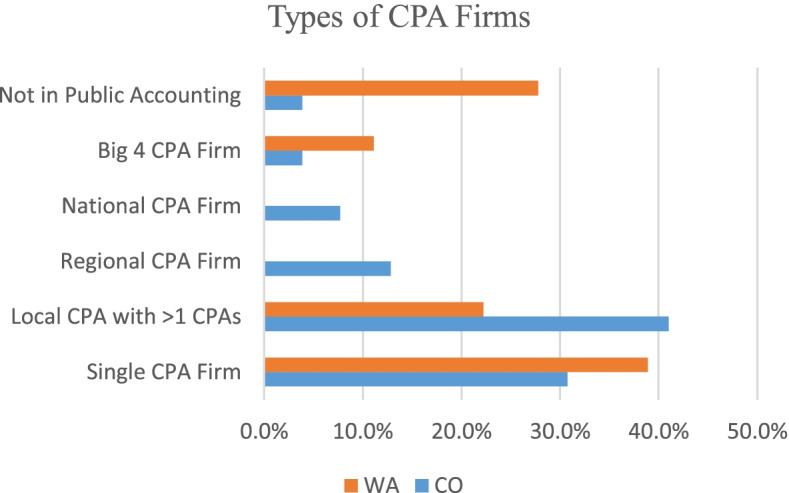
Types of CPA firms reported by survey respondents

Most of the respondents reported employment at small or local CPA firms; Fig. 3 shows the types of firms reported in the survey. This is consistent with the firms of the 885 emailed survey requests; approximately, 17% went to regional, national, and Big Four firms while the majority went to small, local CPA firms. Given that responses were anonymous, it was impossible to know if participants came from email requests or social media requests making the true makeup of the requested population unknown.

### Research question 1: will CPAs serve the industry?

In response to the question “Do you or your firm provide services to a cannabis-related business?” Seventy-seven percent responded that neither they nor their firm provided services to a cannabis-related business client compared to 23% that did serve cannabis clients. A chi-square test indicated that there is no statistically significant difference (*p*-value approximately 1) in proportions of CPAs that will serve the industry between the two states. These accountants primarily provided tax, bookkeeping, and consulting services with audit, litigation support, state compliance/inventory valuation and hemp-only services also mentioned. It is important to note that industrial hemp is no longer federally illegal as it was segregated from cannabis by the Agricultural Improvement Act of 2018. H.R. 2, 115th Cong (2018).

The 23% of CPAs that serve the industry were asked what types of services they provide to CRBs. See Table 1 for detail on the types of services provided to cannabis businesses. Note that respondents could report provision of more than one type of service. It is important to note that tax, bookkeeping, and consulting services may be performed by any accounting professional. Only audit services require the professional to be a licensed CPA. A CRB client needing services is able to use a non-CPA licensed professional for three out of the four services shown in Table 1. Financial statement audit services are generally not required for small businesses unless they are publicly-traded or seeking funding from a commercial source. Many CPA firms avoid providing audit services to any clients due to the risk of legal liability related to financial statement audits. While the CPA license is not required, it is not uncommon for CPA firms to only provide tax, accounting, and consulting services.

**Table 1:** Types of services provided to cannabis-related businesses (CRB) reported by survey respondents

Type of services	Percentage (%)
Tax	90.9
Bookkeeping	59.1
Consulting	45.5
Audit	4.5

Only 13.6% of the 23% of participants that do serve the cannabis industry reported that they have a different fee schedule for CRBs that they described as higher hourly rates due to risk. These 23% of respondents reported client acceptance procedures for CRBs including background checks on owners/management (27%), requiring a retainer for services (45%), requiring an extensive engagement letter (77%), or requiring a personal meeting to establish credibility (5%). Eighteen percent indicated that they had a previous business relationship with the CRB owner and did not require additional client acceptance procedures.

The 23% of participants that do serve the industry responded to an open-ended question that asked what type of special training or technical knowledge they believe is required to service a cannabis-related business. Approximately 64% of these responses included the need for special tax training or training on Internal Revenue Code Section § 280E. Twenty-three percent said that state training or industry-specific training was needed, and 4.5% indicated the need for training and/or knowledge related to internal controls for cash and inventory.

### Research question 2: why are CPAs unwilling to provide services to cannabis-related businesses?

**Fig. 4:**
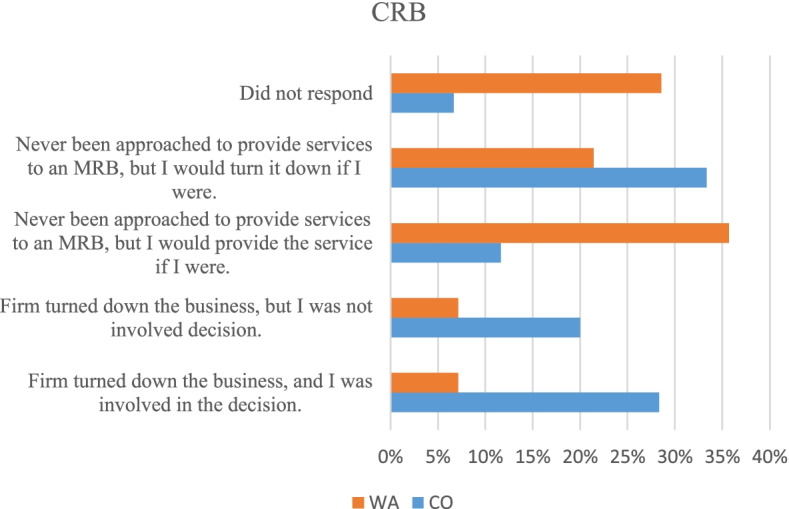
Reasons why CPAs do not serve CRBs reported by survey respondents

Seventy-seven percent of the respondents indicated that neither they nor their firm provide service to CRBs; Fig. 4 indicates why respondents will not serve the industry. For the CPAs shown in Fig. 4, a Pearson’s chi-squared test showed a *p*-value of 0.017 indicating a difference in proportions of reasons given between Colorado and Washington CPAs. More Colorado CPAs were willing to turn down CRB work than were expected, and fewer Colorado CPAs would be willing to take on CRB clients than were expected. While in Washington, fewer CPAs would turn down CRB clients than expected, and more are willing to accept CRB clients than were expected. This could be due to the 2018 Washington State Bill 5928 which stipulates that provision of services to a CRB by a CPA is not a crime (Satterlund 2018). This leads us to believe that Colorado CPAs are not as willing to take on CRB clients as are Washington CPAs.

When provided a list of items that might persuade them to take on a cannabis client, “immunity from federal criminal prosecution” was ranked the highest or second highest by 54% of the CPAs who do not provide services to CRBs. In a similar vein, 35% of CPAs ranked removal of cannabis from the DEA Schedule I Drug listing as number one or two, which would be similar to decriminalizing the industry; this would be enough to persuade them to serve the cannabis industry. Twenty-seven percent of the CPAs ranked “clear support from the state board of accountancy” as either the primary or secondary reason that would persuade them to take on CRB clients. Sixteen percent of CPAs ranked nothing would persuade them to take on cannabis clients as the top response. Table 2 illustrates these results.

**Table 2:** Motivations for CPAs to serve the cannabis industry reported by survey respondents

Response	CPAs ranked as 1 or 2 (most likely to persuade) (%)
Immunity from federal prosecution	54
Removal of cannabis from DEA Schedule I	35
Clear support from the state board of accountancy	27
Nothing	16

### Research question 3: what do CPAs believe is the primary risk related to serving the cannabis industry?

**Fig. 5:**
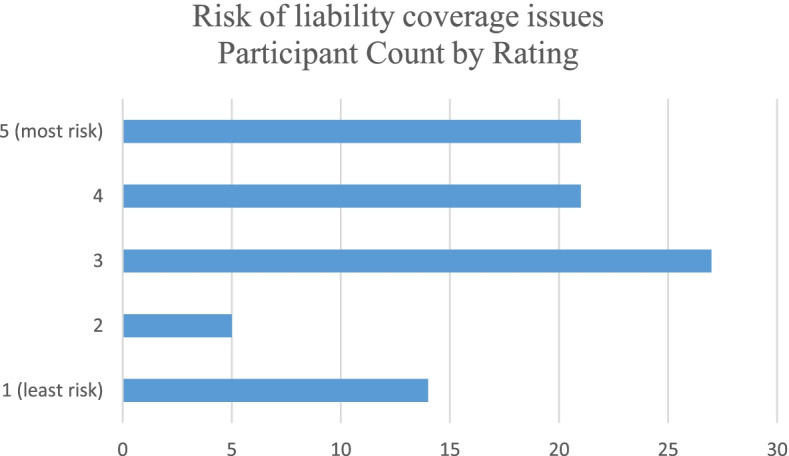
Count of respondents’ ratings (one through five) for risk due to potential professional liability coverage issues

**Fig. 6:**
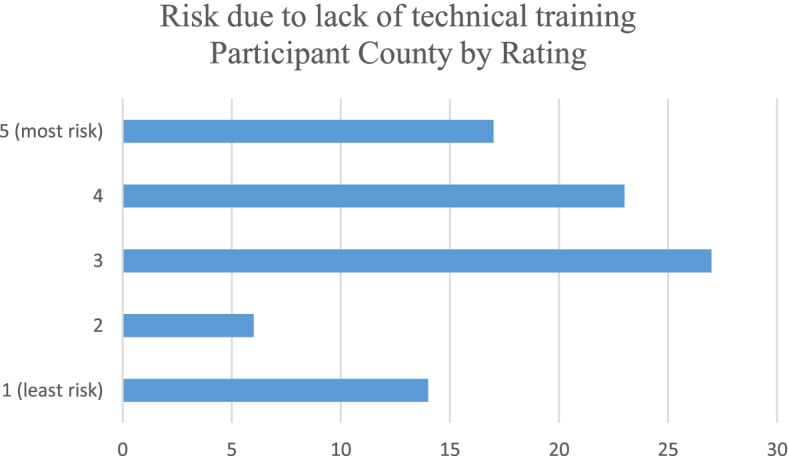
Count of respondents’ ratings (one through five) for risk due to lack of technical industry training

**Fig. 7:**
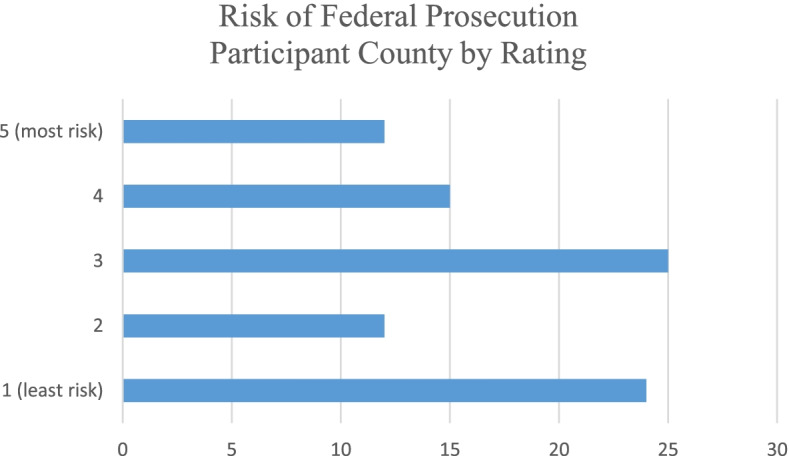
Count of respondents’ ratings (one through five) for risk of federal criminal prosecution

**Fig. 8:**
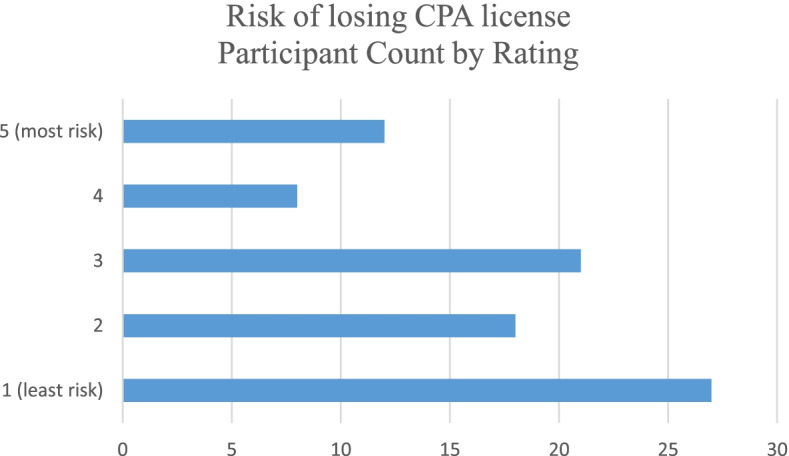
Count of respondents’ ratings (one through five) for risk of losing CPA license

All participants were asked to rate four risks related to providing services to the cannabis industry with 1 being low risk and 5 being high risk. The risk due to potential liability coverage issues was rated the highest with 3.34 while the risk of losing the CPA license was rated lowest at 2.54. Table 3 shows the average ratings per category. Figures 5, 6, 7, and 8 show the counts of ratings one through five by risk type for the respondents. In addition, respondents were asked to list any risks related to providing services to the cannabis industry that were not included in the ranking question. The most common additional risk item mentioned was reputational risk which was indicated by eight respondents. A summary of these responses is reported in Table 4.

**Table 3:** Rating of risk areas according to CPAs responding to the survey. 1 = low risk and 5 = high risk

Risk area	Number of responses^**a**^	Average rating
Risk due to potential professional liability coverage issues	88	3.34
Risk due to lack of technical industry training	87	3.26
Risk of federal criminal prosecution	88	2.76
Risk of losing CPA license	86	2.54

^a^Not all 96 participants responded to these risk questions

**Table 4:** Other potential risk areas reported by participants

Risk area	Number of responses
Reputational risk	8
Risk of working with impaired people/unethical people	5
Cash-intensive business/banking issues	4
Tax/§ 280E complexities/high risk of tax audit	2

The responses to the four risks were analyzed using the Mann-Whitney *U* test (a.k.a. Wilcoxon sum rank test) to determine if significant differences exist between CPAs that serve the industry versus CPAs that do not serve the industry. For three of the four risks, CPAs that do not serve the industry think there is a significantly higher risk than CPAs who do serve the industry. There was no significant difference between the two groups when considering the risk due to lack of technical training to serve the cannabis industry. Table 5 summarizes the test statistics and *p*-values for each risk area.

**Table 5:** Industry risks identified by CPAs who serve vs CPAs who do not serve the industry

Question	Mann-Whitney ***U*** test (***p***-value)
Risk of federal criminal prosecution	438.5 (0.004)
Risk of losing CPA license	347 (0.0003)
Risk due to lack of technical industry training	639.5 (0.45)
Risk due to potential professional liability coverage issues	418.0 (0.0002)

The responses to the four risks were analyzed using the Mann-Whitney *U* test (a.k.a. Wilcoxon sum rank test) to determine if significant differences exist between CPAs from Colorado versus CPAs from Washington. There was no significant difference between CPAs from the two states indicating that CPAs view risk similarly in the two states. Table 6 summarizes the test statistics and p values for each risk area.

**Table 6:** Industry risks identified by CPAs from Colorado vs CPAs from Washington

Question	Mann-Whitney ***U*** test (***p***-value)
Risk of federal criminal prosecution	509 (0.92)
Risk of losing CPA license	476 (0.74)
Risk due to lack of technical industry training	515 (0.96)
Risk due to potential professional liability coverage issues	422 (0.261)

### Research question 4: do CPAs believe serving the cannabis industry would create an ethical issue?

**Fig. 9:**
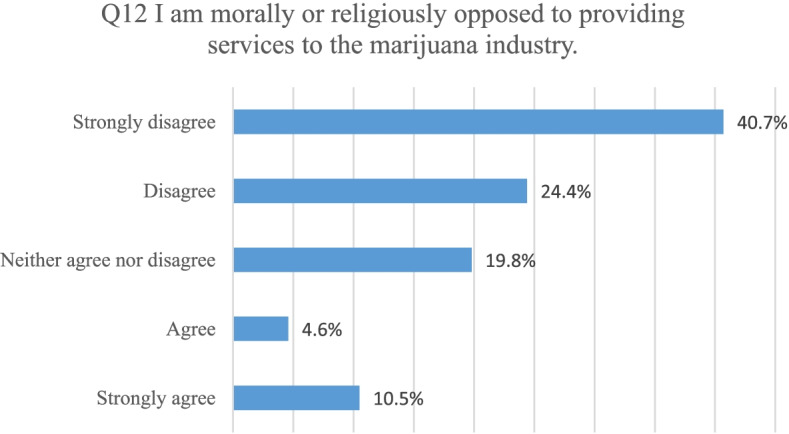
Indication of CPAs’ agreement with the survey statement “I am morally or religiously opposed to providing services to the marijuana industry”

In question 12, all participants were asked to rate their level of agreement with the statement “I am morally or religiously opposed to providing services to the marijuana industry.” Eighty-six of the participants responded to this question. Thirteen participants (15.1%) agreed or strongly agreed. Fifty-six participants (65.1%) disagreed or strongly disagreed. Seventeen participants (19.8%) neither agreed nor disagreed. Figure 9 shows the breakdown of responses for this question.

Question 12 was analyzed to determine if there were differences based on gender, age, or state. No statistical differences were found, leading the researchers to believe that Fig. 6 represents the views of the CPAs in both states.

**Fig. 10:**
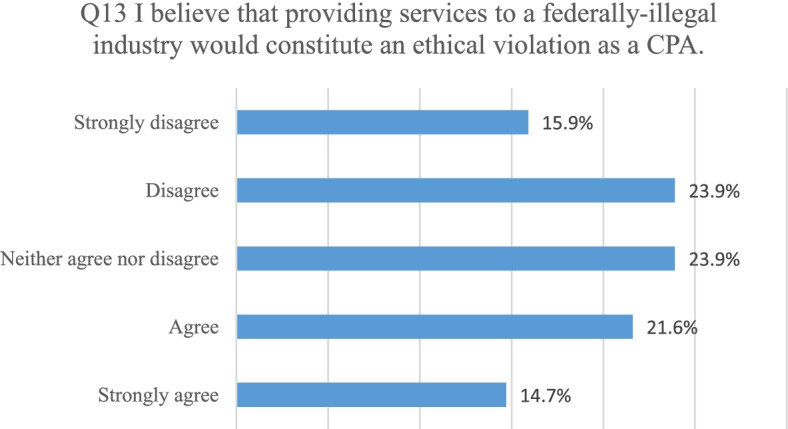
Indication of CPAs’ agreement with the survey statement “I believe that providing services to a federally illegal industry would constitute an ethical violation as a CPA”

In question 13, all participants were asked to rate their level of agreement with the statement “I believe that providing services to a federally-illegal industry would constitute an ethical violation as a CPA.” Eighty-eight of the participants responded to this question. Thirty-two participants (36.3%) agreed or strongly agreed. Thirty-six disagreed or strongly disagreed (39.8%). Twenty-one participants (23.9%) neither agreed nor disagreed. Figure 10 shows the breakdown of responses for this question.

Question 13 was analyzed to determine if there were differences based on gender, age, or state. No statistical differences were found, leading the researchers to believe that Fig. 10 represents the views of the CPAs in both states.

## Discussion

Research question 1: The data found that, in fact, most CPAs surveyed (74.3%) do not serve the cannabis industry. This data is consistent with the Owens-Ott (2020) study in which 64.3% of participants were unwilling to serve the industry. This belief is confirmed by Tables 2, 3, and 5 where CPAs surveyed were concerned with the potential legal ramifications of servicing CRBs and potential risk of professional liability. However, as confirmed by Figs. 5 and 6, while CPAs themselves are not personally (morally or religiously) opposed to servicing CRBs, many feel that a professional or legal ethical issue might compromise their standing in the profession or put at risk their professional license.

Research question 2: Why are CPAs unwilling to provide services to cannabis-related businesses? Most CPAs indicated that immunity from federal criminal prosecution (54.1%) or removal from the DEA Schedule I Drug Listing (35.1%) would motivate them to take on a CRB. A few CPAs (27.0%) indicated that clear support from the state board of accountancy would persuade them to take on CRB clients. Approximately 16% said nothing would persuade them to take on cannabis clients. Table 7 compares results from this study to the Owens-Ott (2020) study.

**Table 7:** Comparison of reasons CPAs will not serve the industry from this study and the 2020 Owens-Ott study

Reasons CPAs will not serve the industry	Current study (%)	Owens-Ott (2020) study (%)
Possible federal criminal prosecution/immunity from federal prosecution/removal of cannabis from DEA Schedule I	89.2	89
Fear of losing CPA license/clear support from state board of accountancy	27.0	33
Complicated tax requirements; not willing to devote the time and resources to special technical training	17.6	44

While there appears to be a noticeable difference between the two studies regarding why CPAs in this study were less concerned with complicated tax requirements or need for special technical training than the previous study, that is more likely due to differences in the way participants were questioned. The original study allowed participants to indicate more than one reason they would not serve the industry without requiring them to rank the responses. This study had participants rank reasons to not serve the industry and Table 7 shows percentages of participants that ranked each reason as one or two. However, 47% of participants ranked the complicated tax requirements as three or four (out of eight potential reasons) indicating they were still concerned about it, just not as much as federal prosecution.

Research question 3: What do CPAs believe is the primary risk related to serving the cannabis industry? Respondents rated risk related to professional liability insurance coverage and risk because of a lack of technical training as the highest risk factors. Risk of federal criminal prosecution and risk of losing CPA license were ranked lower by participants. Table 8 compares the risks reported in this study compared to the Owens-Ott (2020) study. The primary difference was that participants in this study rated the risk related to professional liability issues as the highest and was rated lower in the Owens-Ott (2020) study.

**Table 8:** Comparison of risks CPAs identified from this study and the 2020 Owens-Ott study

Risks related to serving the industry	Current Study (percentage of participants who indicated 4 or 5 out of 5) (%)	Owens-Ott (2020) study (percentage identified risk) (%)
Professional liability insurance issues/litigation	48	14
Lack of technical training/complicated tax code	46	29
Federal criminal prosecution	30	29
Risks of losing CPA license	23	29

A Wilcoxon rank sum test showed that CPAs who do not serve the industry express higher risk assessment for federal criminal prosecution than CPAs who do serve the industry (*p*-value = 0.004). A Wilcoxon rank sum test showed that CPAs who do not serve the industry ranked risk of losing the CPA license higher than CPAs who do serve the industry (*p*-value = 0.0003). A Wilcoxon rank sum test showed that CPAs who do not serve the industry ranked risk liability insurance coverage issues higher than CPAs who do serve the industry (*p*-value = 0.0002). There was no statistically significant difference between the two groups for the risk due to lack of technical training.

Research question 4: Do CPAs believe serving the cannabis industry would create an ethical issue? Participants provided insights on their ethical perspectives related to serving the cannabis industry. The last two survey questions were devoted to understanding these perspectives. Most CPAs surveyed indicated they were not morally or religiously opposed to providing services to cannabis businesses. Participants were fairly evenly divided on whether they viewed providing services to a federally illegal business as an ethical violation for a CPA. It remains unclear to CPAs if this would be an ethical violation according to the AICPA Code of Conduct as the Code does not specifically allow or disallow CRB engagements. Data indicated that there was not a statistically significant difference between Colorado and Washington participants related to whether they were morally or religiously opposed to working in the industry or if they viewed serving the industry as an ethical violation.

## Limitations

The scope of this study was limited to Colorado and Washington CPAs. The perspectives of the participants may not necessarily reflect the perspectives of all CPAs in Colorado or Washington or be generalizable to other geographic areas. CPAs in states with less mature cannabis markets may have different perspectives than CPAs surveyed in these two mature market states. While the total number of respondents was acceptable, there were far fewer usable responses from Washington CPAs than from Colorado CPAs. The researchers attempted to draft questions in a neutral tone and to analyze data in an unbiased manner to avoid introducing unconscious bias into the study.

## Recommendations for future research

More could be learned about the perspectives of CPAs in Colorado and Washington. A larger sample size would be useful to determine if there are indeed differences in gender, age groups, states, and types of CPA firms. In addition, responses of CPAs in these two mature cannabis states might not be consistent with CPAs in states with less mature cannabis markets. Additional data could be gathered from CPAs in a wide variety of states to determine if the perspectives are similar to those found in this study or not. It may also be interesting to apply the theories of organizational stigma and stigma transfer to this research. The authors are pursuing a follow-up study to investigate the perspectives of future CPAs/university accounting students compared to current CPAs.

## Conclusion

Consistent with the 2019 qualitative study and previous research, CPAs do remain largely unwilling to serve the cannabis industry. The reason is primarily that CPAs fear federal prosecution as long as cannabis remains on the DEA’s Schedule I Drug Listing. The results of this study indicate that while most CPAs are not morally or religiously opposed to serving the industry, respondents are evenly split on whether doing so may constitute an ethical violation for a CPA.

## Data Availability

Data can be made available upon request.

## Appendix

Survey Questions for CPAs

Survey questions for CPAs licensed in Colorado and Washington (administer via Survey Monkey):

1. Demographic and firm information:

- Select your gender

□ Male

□ Female

□ Prefer not to answer

b. Indicate your age
  □ ______
  □ Prefer not to answer
c. In what state(s) do you hold a CPA license? (select all that apply)
  □ CO
  □ WA

□ I’ve passed the CPA exam, but am not yet licensed

□ Other: Please list

d. In what zip code do you primarily practice?
  _ _ _ _ _
e. Which of the following best describes your CPA firm?
  □ Single CPA
  □ Local CPA firm with multiple CPAs
  □ Regional CPA firm
  □ National CPA firm
  □ Big 4 CPA firm
  □ Not in public accounting

2. Do you or your firm provide services to a marijuana-related business (MRB)? If yes, go to question 3. If no, skip to question 9.
  □ Yes (go to question 3)
  □ No (go to question 11)
3. What types of services do you provide to MRBs? Select all that apply:
  □ Tax
  □ Bookkeeping
  □ Audit
  □ Consulting (describe)_______________________________________________
  □ Other (describe)___________________________________________________
4. Do you have a different fee schedule for MRBs?
  □ Yes: (Describe) ____________________________________________________
  □ No
  □ Unsure
5. What types of client acceptance procedures did you perform prior to accepting the MRB client? Select all that apply:
  □ Background checks on owners/management
  □ Require a retainer for services
  □ Require extensive engagement letter
  □ Other (Describe)_____________________________________________________
6. What type of special training or technical knowledge do you believe is required to service the MRB? (Open ended question)
7. Are there any services that you choose NOT to provide to MRBS?
  □ No
  □ Yes (what services and why?)
8. Rate each of the following in terms of your perception of risk related to providing services to MRBs with 5 being highly risky and 1 being no risk:

- Federal criminal prosecution
  5 High risk
  4 Significant risk
  3 Risk
  2 Low risk
  1 Not a risk

b. Losing CPA license
  5 High risk
  4 Significant risk
  3 Risk
  2 Low risk
  1 Not a risk

c. Lack of technical industry knowledge to competently provide service to MRBs
  5 High risk
  4 Significant risk
  3 Risk
  2 Low risk
  1 Not a risk

d. Professional liability insurance coverage concerns
  5 High risk
  4 Significant risk
  3 Risk
  2 Low risk
  1 Not a risk

END SURVEY FOR THOSE WHO ANSWERED YES ON NUMBER 2

REMAINDER OF SURVEY FOR THOSE WHO ANSWERED NO ON NUMBER 2:

9. Why do you not provide service to MRBs?
  □ Never been approached to provide services to an MRB, but I would turn it down if I were.
  □ Never been approached to provide services to an MRB, but I would provide the service if I were.
  □ Firm turned down the business, but I was not involved decision.
  □ Firm turned down the business, and I was involved in the decision.
10. Rate each of the following in terms of the likelihood that it would persuade you to take on MRBs with 5 being highly likely and 1 being highly unlikely:
  □ Immunity from federal criminal prosecution
  □ Clear support from the state board of accountancy
  □ Industry guide on accounting and tax matters
  □ Guidance on risk assessment procedures
  □ Removal of marijuana from the DEA Schedule I Drug listing
  □ Significantly higher fee structure
  □ Support from other CPAs within my firm
  □ Nothing
  □ Other, please describe_______________________________________
11. Rate each of the following in terms of your perception of risk related to providing services to MRBs with 5 being highly risky and 1 being no risk:

- Federal criminal prosecution
  5 High risk
  4 Significant risk
  3 Risk
  2 Low risk
  1 Not a risk

b. Losing CPA license
  5 High risk
  4 Significant risk
  3 Risk
  2 Low risk
  1 Not a risk

c. Lack of technical industry knowledge to competently provide service to MRBs
  5 High risk
  4 Significant risk
  3 Risk
  2 Low risk
  1 Not a risk

d. Professional liability insurance coverage concern
  5 High risk
  4 Significant risk
  3 Risk
  2 Low risk
  1 Not a risk

12. I am morally or religiously opposed to working with MRBs.
  5 Strongly agree
  4 Agree
  3 Neither agree nor disagree
  2 Disagree
  1 Strongly disagree

13. I believe that providing services to a federally illegal industry would constitute an ethical violation as a CPA.
  5 Strongly agree
  4 Agree
  3 Neither agree nor disagree
  2 Disagree
  1 Strongly disagree

## Abbreviations

CPA: Certified public accountant
DEA: Drug Enforcement Agency
AICPA: American Institute of Certified Public Accountants
BOA: Board of Accountancy
CRB: Cannabis-related business

## References

[CR1] Agricultural Improvement Act of 2018. H.R. 2, 115th Cong. (2018). Retrieved from: https://www.congress.gov/bill/115-congress/house-bill/2. Accessed 23 Oct 2020.

[CR2] AICPA. (2014). Code of professional conduct. Retrieved from AICPA Online Professional Library: https://pub.aicpa.org/codeofconduct/Ethics.aspx.

[CR3] AICPA. (2016. An issue brief on state marijuana laws and the CPA profession. Retrieved from AICPA: https://www.aicpa.org/Advocacy/State/DownloadableDocuments/MarijuanaCPAsIssueBrief.pdf.

[CR4] Bryman A, Bell E (2011). Business research methods.

[CR5] Cheon H, Decker SH, Katz CM (2018). Medical marijuana and crime: substance use and criminal behaviors in a sample of arrestees. J Drug Issues.

[CR6] Chiang WC, Du J, Summers D. Providing services to the marijuana industry. CPA J. 2019; Retrieved from: https://www.cpajournal.com/2019/11/29/providing-services-to-the-marijuana-industry/.

[CR7] Colorado Board of Accountancy (2015). Colorado Board of Accountancy’s position statement regarding certified public accountant certificate holder’s providing service to the marijuana industry. Retrieved from https://drive.google.com/file/d/0BzKoVwvexVATeHg4NE5SV29zaW8/view.

[CR8] Creswell JW, Creswell JD (2018). Research design: qualitative, quantitative, and mixed methods approaches.

[CR9] Devers CE, Dewett T, Mishina Y, Belsito CA (2009). A general theory of organizational stigma. Organ Sci.

[CR10] Doherty C, Tyson A, Weisel R. In debate over legalizing marijuana, disagreements over drug’s dangers. 2015; Retrieved from Pew Research Center: http://www.people-press.org/2015/04/14/in-debate-over-legalizing-marijuana-disagreement-over-drugs-dangers/.

[CR11] Hampel CE, Tracey P (2016). How organizations move from stigma to legitimacy: the case of Cook’s travel Agency in Victorian Britain. Acad Manag J.

[CR12] Hudson BA, Okhuysen GA (2009). Not with a ten-foot pole: core stigma, stigma transfer, and improbably persistence of men’s bathhouses. Organ Sci.

[CR13] Miles MB, Huberman AM (1994). Qualitative data analysis: an expanded sourcebook.

[CR14] MPG Consulting & Leeds School of Business. (2019). 2019 Regulated Marijuana Market Update Report Final. Retrieved from https://www.colorado.gov/pacific/sites/default/files/2019%20Regulated%20Marijuana%20Market%20Update%20Report%20Final.pdf

[CR15] NASBA. (2019). Providing services to businesses in the marijuana industry: a sample of current board positions. Retrieved from https://www.aicpa.org/content/dam/aicpa/advocacy/state/downloadabledocuments/marijuana-state-board-positions.pdf.

[CR16] National Conference of State Legislatures (2020. State medical marijuana laws. Retrieved from: https://www.ncsl.org/research/health/state-medical-marijuana-laws.aspx. Accessed 3 Sep 2020.

[CR17] Owens-Ott GS (2020). Accounting and the US cannabis industry: federal financial regulations and the perspectives of certified public accountants and cannabis businesses owners. J Cannabis Res.

[CR18] Pew Research Center. (2015). In debate over legalizing marijuana, disagreement over drug’s dangers. Retrieved from Pew Research Center website: http://www.people-press.org/2015/04/14/in-debate-over-legalizing-marijuana-disagreement-over-drugs-dangers/.

[CR19] Polonsky MJ, Waller DS (2005). Designing and managing a research project: a business student's guide.

[CR20] Robinson F (2017). Going green: legal considerations for marijuana investors and entrepreneurs. Am Univ Bus Law Rev.

[CR21] Satterlund, C. (2018). CPAs and cannabis. Retrieved from Board of Accountancy Washington State: https://acb.wa.gov/cpas-and-cannabis.

[CR22] Sterna S, Wolfe J. Liability and other concerns when servicing marijuana businesses. CPA J. 2017:9–10.

[CR23] Taylor K, Bunker RB, Johnson LR, Rodriguez R (2016). An analysis of the accounting and financial effects of inconsistent state and federal laws in the recreational cannabis industry. J Legal Ethical Regul Issues.

[CR24] Thomas G (2009). How to do your research project: a guide for students in education and applied social sciences.

